# The Emergence of SARS-CoV-2 within the Dog Population in Croatia: Host Factors and Clinical Outcome

**DOI:** 10.3390/v13081430

**Published:** 2021-07-22

**Authors:** Vladimir Stevanovic, Irena Tabain, Tatjana Vilibic-Cavlek, Maja Mauric Maljkovic, Iva Benvin, Zeljka Hruskar, Snjezana Kovac, Iva Smit, Gorana Miletic, Suzana Hadina, Vilim Staresina, Lada Radin, Valentina Plichta, Branimir Skrlin, Zoran Vrbanac, Mirna Brkljacic, Marija Cvetnic, Josipa Habus, Kresimir Martinkovic, Iva Zecevic, Gabrijela Jurkic, Ivana Ferencak, Zinka Stritof, Matko Perharic, Lovro Bucic, Ljubo Barbic

**Affiliations:** 1 Department of Microbiology and Infectious Diseases with Clinic, Faculty of Veterinary Medicine, University of Zagreb, 10000 Zagreb, Croatia; iva.benvin@vef.hr (I.B.); snjezana.kovac@vef.hr (S.K.); gogamiletic008@gmail.com (G.M.); vilim.staresina@vef.hr (V.S.); ljubo.barbic@vef.hr (L.B.); 2 Veterinary Teaching Hospital, Faculty of Veterinary Medicine, University of Zagreb, 10000 Zagreb, Croatia; iva.smit@vef.hr (I.S.); suzana.hadina@vef.hr (S.H.); lada.radin@vef.hr (L.R.); valentina.plichta@vef.hr (V.P.); branimir.skrlin@vef.hr (B.S.); zoran.vrbanac@vef.hr (Z.V.); mirna.brkljacic@vef.hr (M.B.); marija.cvetnic@vef.hr (M.C.); josipa.habus@vef.hr (J.H.); kresimir.martinkovic@vef.hr (K.M.); iva.zecevic@vef.hr (I.Z.); gjurkic@vef.hr (G.J.); zrinka.stritof@vef.hr (Z.S.); matko.perharic@vef.hr (M.P.); 3 Department of Virology, Croatian Institute of Public Health, 10000 Zagreb, Croatia; zeljka.hruskar@hzjz.hr (Z.H.); ivana.ferencak@hzjz.hr (I.F.); 4 School of Medicine, University of Zagreb, 10000 Zagreb, Croatia; 5 Department for Animal Breeding and Livestock Production, Faculty of Veterinary Medicine, University of Zagreb, 10000 Zagreb, Croatia; maja.mauric@vef.hr; 6 Department of Epidemiology, Croatian Institute of Public Health, 10000 Zagreb, Croatia; lovro.bucic@hzjz.hr

**Keywords:** SARS-CoV-2, animals, dogs, epidemiology, risk factors, clinical picture, Croatia

## Abstract

Over a year into the COVID-19 pandemic, there is growing evidence that SARS-CoV-2 infections among dogs are more common than previously thought. In this study, the prevalence of SARS-CoV-2 antibodies was investigated in two dog populations. The first group was comprised of 1069 dogs admitted to the Veterinary Teaching Hospital for any given reason. The second group included dogs that shared households with confirmed COVID-19 cases in humans. This study group numbered 78 dogs. In COVID-19 infected households, 43.9% tested ELISA positive, and neutralising antibodies were detected in 25.64% of dogs. Those data are comparable with the secondary attack rate in the human population. With 14.69% of dogs in the general population testing ELISA positive, there was a surge of SARS-CoV-2 infections within the dog population amid the second wave of the pandemic. Noticeably seroprevalence of SARS-CoV-2 in the dog and the human population did not differ at the end of the study period. Male sex, breed and age were identified as significant risk factors. This study gives strong evidence that while acute dog infections are mostly asymptomatic, they can pose a significant risk to dog health. Due to the retrospective nature of this study, samples for viral isolation and PCR were unavailable. Still, seropositive dogs had a 1.97 times greater risk for developing central nervous symptoms.

## 1. Introduction

Animal origin of severe acute respiratory syndrome coronavirus 2 (SARS-CoV-2) has been hypothesised since the beginning of the coronavirus disease (COVID-19) pandemic [1]. Under experimental conditions, SARS-CoV-2 has a broad range of susceptible animal hosts [2,3]. Due to close contact with owners, pet animals were assumed to be at increased risk of infection in natural conditions. As opposed to the results of experimental infections [4], early studies showed that in natural settings probability of dogs getting infected is the same or higher than in cats [5,6,7,8]. 

There is currently no evidence that dogs have a significant role in the virus spreading. On the other hand, the rising number of confirmed infections in dogs raises questions regarding the clinical consequences because the clinical spectrum of COVID-19 in humans is still being updated. An additional concern is rising over post-acute COVID-19 syndrome with a long-lasting effect on health [9]. 

This study aimed to examine the prevalence of SARS-CoV-2 infection in the Croatian dog population and host factors predisposing to infection. The study was conducted in two populations, dogs from COVID-19 infected households and those admitted to the Veterinary Teaching Hospital. Two serological tests were used, and their sensitivity and specificity were determined. The medical history and clinical presentation data were analysed to address the potential clinical manifestation of SARS-CoV-2 infections in dogs.

## 2. Materials and Methods

On 18 December 2020, dog owners diagnosed with COVID-19 were invited to join this study. Only owners with positive RT-PCR tests could enter the study. After signing the consent, signalment, dog medical history and blood samples were taken. Obtained serum samples were stored at −80 °C until testing. A total of 78 dogs were part of this study.

From 1 July till 31 December 2020, remaining dog serum samples were collected. Dogs were sampled during their healthcare visits at Veterinary Teaching Hospital at the Faculty of Veterinary Medicine, University of Zagreb, Croatia (Veterinary Hospital). To be included in this study, signalment data such as breed, sex, age and place of residence had to be available. For statistical analysis, medical history data were collected as well. In total, 1069 dog serum samples and their data were included in the study. In the following text, those dogs are referred to as the general population. 

A total of 169 serum samples collected during 2018 and 2019 were used as the negative control. The general population was represented with an age-stratified panel of 104 serum samples. Additionally, 65 randomly selected samples of dogs from breeding colonies were included. 

Medical records of 1000 randomly selected dogs admitted to University Hospital from 1 July till 31 December 2019 were retrieved. The inclusion criterion was that dogs were blood sampled during a veterinary visit. 

A sampling of animals and retrieving medical data for this study was approved by the Ethics Committee of the Faculty of Veterinary Medicine, the University of Zagreb, Croatia (decision number: 640-01/20-02/12).

Detection of SARS-CoV-2 antibodies was performed by an indirect enzyme-linked immunosorbent assay (ELISA). The receptor-binding domain (RBD) of SARS-CoV-2 strain Wuhan-Hu-1spike protein (NCBI Reference Sequence: YP_009724390.1) was used for this test. Each serum sample was tested in two wells, one coated with RBD protein solution and the other with phosphate-buffered saline (PBS) to avoid false-positive results due to serum-specific background noise [10]. Designated wells of ELISA plate (Nunc MaxiSorp, Thermo Fisher Scientific, Waltham, MA) were coated with 100 ng of RBD (Sino Biological, Beijing, China) diluted in 50 µL of PBS or the same volume of PBS. Following overnight incubation, the plate was washed twice with 300 µL of PBS per well using an automatic ELISA plate washer (Tecan, Männedorf, Switzerland). Blocking was done by adding 200 µL of 3% bovine serum albumins (St. Louis, MO, USA) and 0.5% (v/v) Tween 20 (Santa Cruz Biotechnology, Santa Cruz, CA, USA) in PBS in all wells and incubation for 2 h at 37 °C. Subsequently, wells were washed three times with 300 µL of washing buffer made of PBS and 0.5% Tween 20. Serum samples diluted in blocking buffer (1:100) were added in one RBD coated well and one well coated with PBS, in the volume of 100 µL. After one hour of incubation at room temperature, the plate was washed five times. Horseradish peroxidase-conjugated goat anti-dog IgG antibodies (Jackson ImmunoResearch Europe, Ely, United Kingdom) were diluted 1:65000 in blocking buffer and added 100 µL per well. After one hour at room temperature, the plate was washed five times. In the next step, 100 µL of tetramethylbenzidine substrate (TMB High Sensitivity ELISA substrate) (Abcam, Cambridge, United Kingdom) per well was added. After 15 min of incubation in the dark, the reaction was stopped by adding the same volume of 1M H_2_SO_4_ (Kemika, Zagreb, Croatia). Optical density (OD) was read on the microplate reader (Tecan, Männedorf, Switzerland) at 450 nm. Corrected OD for each sample was calculated as a difference of OD between RBD coated and non-coated well. The initial cut-off value was calculated by adding three standard deviations to the arithmetic mean of corrected OD values of 169 control samples. Samples were regarded as positive if corrected OD > 0.203. In subsequent runs, every plate had positive, negative and cut-off control included. 

The microneutralisation test has been described previously [6]. In brief, serial twofold dilutions of serum samples were prepared in wells of a 96-well microneutralisation plate. Each dilution was tested in duplicate, mixing 25 µL of serum dilution and the same volume of SARS-CoV-2 virus containing 100 TCID_50_ viral particles. The results’ repeatability was ensured by virus back titration and low titre positive and negative dog serum samples included in each run. After one hour of incubation at 37 °C with CO_2_, 50 µL of 2 × 10^5^ E6 Vero cells/mL were added to each well. Antibody titre was the reciprocal of the highest dilution of the serum that showed 100% neutralisation after four days of incubation. MNT procedure was carried out in the biosecurity level 3 laboratory. Ten known CCoV and CRCoV positive pre-pandemic serum samples were tested to assess MNT specificity. Due to some samples’ cytotoxicity, a serum was defined as MNT positive if the antibody titre was 1:8 or more.

All control samples were pretested for the canine respiratory coronavirus (CRCoV) antibodies. Most CRCoV serological testing methods utilise a high degree of similarity between CRCoV and bovine coronavirus (BCoV) spike proteins and resulting serological crossreactivity [11]. This study used a commercial ELISA kit (BIO K 392 Monoscreen AbELISA Bovine coronavirus/Competition, Bio-X diagnostics, Rochefort Belgium) as previously described [12]. It is a competitive ELISA with wells coated with BCoV antigens. Samples were tested according to the manufacturer’s instructions using positive and negative controls provided in the kit.

For 40 control serum samples, canine coronavirus (CCoV) antibody status was determined as well. Samples were tested following the manufacturer’s instructions provided in the commercial ELISA kit Ingenzim Corona Canino 15.CCV.K1 (Ingenasa, Madrid, Spain).

Descriptive statistics are presented as numbers and percentages. All statistical analysis was performed using R 4.0.5. Seroprevalence rates and exact 95% confidence intervals (95% CI) were calculated using epiR. Obtained data were analysed using the two-tailed χ^2^ test or Fishers’ exact test, and p values below 0.05 were considered statistically significant. The odds ratio (OR) of bivariate risk factors, risk ratio (RR), and 95% CI were calculated using epitools. Logistic regression analysis was used to calculate OR of multinomial risk factors and assess interactions between risk factors. The multivariable logistic regression model was selected through the Akaike information criterion when evaluating interactions between risk factors. The logit link in glm was used, and NA (not available) were excluded from the model. In the variable breed clades, complete separation occurred, so the Firth correction was applied using logistf. Association between ELISA and MNT was determined with Cohen’s kappa coefficient (κ) calculated using irr. To calculate the strength of association between ELISA’s corrected OD (continuous variable) and MNT result (binary variable) the Point-Biserial correlation coefficient (r_pb_) was used. Spearman’s rho correlation coefficient (rho) value represented the association between ELISA’s corrected OD and MNT antibody titre (analysed as the logarithmic value of antibody titre) was represented by Spearman’s rho correlation coefficient (rho) value. The age structure of dog populations in 2019 and 2020 was compared by the Mann–Whitney U test (MWU).

## 3. Results

### 3.1. Methods and Seroprevalence

Pre-pandemic samples were tested to exclude potential cross-reactivity of SARS-CoV-2 antigens and antibodies against CCoV (alphacoronavirus) and CRCoV (betacoronavirus). In the control group, 41.3% (*n* = 104) were positive for CRCoV, and 27 samples were known positive for CCoV antibodies, but none of the pre-pandemic samples tested ELISA positive. The same was true for the neutralisation assay. In COVID-19 positive households, ELISA reactivity was detected in 43.9%, and neutralising antibodies were detected in 25.64% of dogs (*n* = 78). There was a substantial agreement of methods with a kappa value of 0.62 (*n* = 78, 95% CI 0.45–0.75). There was a statistically significant correlation between OD value and the result of MNT (*n* = 34, r_pb_ = 0.52, 95% CI 0.22–0.73, *p* = 0.002) and OD value and neutralisation titre (*n* = 20, rho = 0.58, 95% CI 0.28–0.8, *p* = 0.0003). In the general population, 157 (14.69%, *n* = 1069) samples were ELISA positive, and those samples were MNT tested. For eight sera, there was not enough remaining volume. Out of 149 ELISA positive samples, 23 had neutralising activity. The kappa value of 0.24 (*n* = 1038, 95% CI 0.16–0.32) showed fair agreement of MNT and ELISA. Once again, there was a significant correlation of the MNT results and calculated OD value (*n* = 148, r_pb_ = 0.61, 95% CI 0.49–0.7, *p* < 0.0001) as well as neutralisation titre an OD (*n* = 148, rho = 0.51, 95% CI 0.18–0.77, *p* = 0.01).

**Figure 1:**
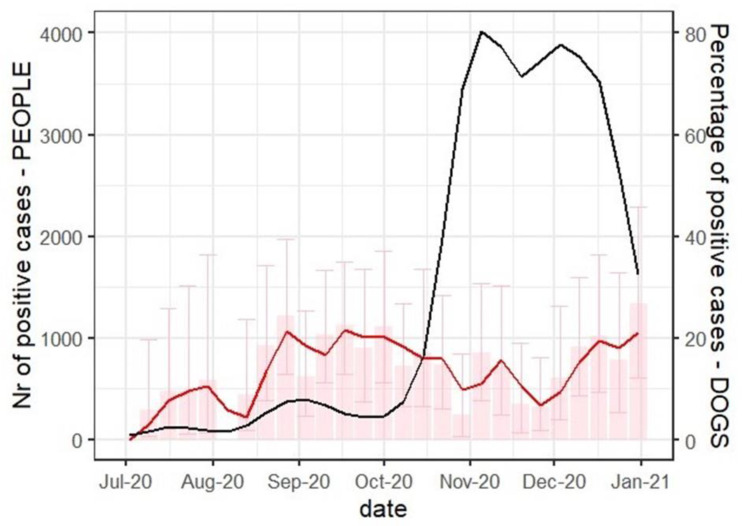
The weekly number of SARS-CoV-2 seropositive dogs and new q-RT PCR human cases in Zagreb from 1 July and 31 December 2020. The number of seropositive dogs is represented as a percentage of tested samples (red) and a two-week moving average trend line (red). Error bars are representing a 95% confidence interval. The number of new human SARS-CoV-2 infections in Zareb is publicly available [13] and here is represented as a two-week moving average trend line in black.

The positivity rate of ELISA tested samples varied significantly, with the lowest prevalence in July (*n* = 126, 7.14%, 95% CI 3.32–13.13) and highest in September (*n* = 233, 19.74%, 95% CI 14.83–25.44) (Table 1, Figure 1). Out of 23 ELISA positive samples which showed neutralisation activity, 16 were collected in December (*n* = 149, Fisher’s exact test *p* < 0.0001). 

### 3.2. Predisposing Factors

Living in COVID-19 positive households was a significant risk factor for seroconversion. The odds of testing ELISA positive were 4.49 (*n* = 1147, OR 95% CI 2.78–7.24, *p* < 0.0001) times higher for dogs in infected households than dogs in the general population. In other words, dogs from COVID-19 infected households had a 2.97 times greater risk of infection than in the general population (*n* = 1147, RR 95% CI 2.22–3.97, *p* < 0.0001). In positive households, ELISA positive dogs were 7.83 (*n* = 183, OR 95% CI 3.46–17.68 *p* < 0.0001) times more likely to test MNT positive than in the general population. 

On the other hand, living in animal shelters was not a significant risk factor. The rate of ELISA positivity was similar in the general population between privately owned and dogs from shelters (*n* = 1065, OR = 1.03, 95% CI 0.35–3.01, *p* = 1).

Another important predisposing factor was sex. In dogs from COVID-19 positive households, female dogs were overrepresented with 63.63% (*n* = 77). Male dogs tested were more often ELISA positive, but the difference did not mount to the level of statistical significance (*n* = 77, OR = 1.16, 95% CI 0.45–2.94, *p* = 0.76). MNT results were similar, with 28.57% male testing positive compared to 24.49% female animals (*n* = 77, OR = 1.23, 95% CI 0.43–3.51, *p* = 0.69). In the general population, the female to male ratio was more even. Male dogs were 1.6 times more likely to be ELISA positive than female (*n* = 1069, OR = 1.6, 95% CI 1.13–2.27 *p* = 0.008). Results of the MNT testing were not influenced by sex (*n* = 149, OR = 1.45, 95% CI 0.56–3.79, *p* = 0.44).

The age of dogs was from few months up to 15 years in positive households and 18 years in the general population. Among dogs from COVID-19 infected households lowest seroprevalence was recorded in dogs under one year of age, with a significantly increased positivity rate in dogs aged between one and five years (*n* = 75, OR = 10.5, 95% CI 1.44–27.82, *p* = 0.04). In this group, the number of dogs with neutralising antibodies did not significantly differ between age groups (Supplementary Table S1). In the general population, dogs aged five and six years were most likely to test ELISA positive (*n* = 1069, OR = 1.67, 95% CI 1.02–2.17 *p* = 0.038) (Supplementary Figure S1). When age distribution in the general population was compared between male and female dogs, odds of male dogs between four and seven years of age to test ELISA positive were 2.11 (*n* = 555, OR = 2.11, 95% CI 1.25–3.54, *p* = 0.0048) times greater than males of any other age and 3.82 (*n* = 171, OR = 3.82 95% CI 1.56–9.39, *p* = 0.0034) times greater than females of the same age. Dogs aged five and six years were also most likely to test MNT positive (*n* = 145, OR = 3.15, 95% CI 1.11–8.92, *p* = 0.048). Due to the relatively small number of samples, ELISA positive dogs were classified into age groups corresponding to COVID-19 infected households. There was no statistically significant difference in MNT positivity rate (*n* = 145, Fisher’s exact test *p* = 0.89). 

Among dogs from COVID-19 infected households, 29 breeds and a mixed-breed group were represented, and in the general population, 89 breeds and a mixed-breed group were represented. In dogs from COVID-19 infected households, the sample size was too small to further address breed predisposition to SARS-CoV-2 infection. In the general population, a small number of animals represented many breeds, so purebred dogs were further grouped into the clades based on their phylogenetic relationship [14]. When assessing breed clades, with the mixed breed as the reference category, only the Continental Herders stood out significantly different (OR = 4.63 95% CI 1.94–11.03 *p* = 0.0005) (Supplementary Table S2). Belgian Shepherds only represented this clade with 3.19 times greater risk of testing ELISA positive than any other breed (*n* = 707, RR 95%CI 1.84–5.51, *p* < 0.001). Upper respiratory system disorders have been associated with the brachycephalic skull conformation [15,16]. Brachycephalic dogs did not test more ELISA positive (*n* = 739, OR = 1.02, 95% CI 0.66–1.58, *p* = 0.94) or MNT positive (*n* = 95, OR = 0.56, 95% CI 0.14–2.22, *p* = 0.53).

### 3.3. Clinical Manifestation

Dog owners from COVID positive households did not notice any apparent change in their pets’ health. In the general population, clinical information was retrieved from their medical records and validated by the clinicians. 

Based on the anamnesis, initial presentation, physical examination and X-ray, dogs were classified into two large groups: animals with signs of respiratory system involvement and animals with signs of digestive system involvement, regardless of the underlying condition. Neither animals with respiratory (*n* = 728, OR = 0.62, 95% CI 0.37–1.04, *p* = 0.07) nor animals with gastrointestinal symptoms (*n* = 1036, OR = 0.87, 95% CI 0.56–1.35, *p* = 0.53) were more likely ELISA positive. The clinical manifestations were further classified into acute respiratory or gastrointestinal disorders, chronic respiratory or gastrointestinal disorders, central nervous system (CNS), ophthalmic conditions, dermatologic disorders, heart disorders, malignancies, endocrine disorders, surgical cases, general health check, nonspecific signs of early infection, and other disorders. The last group included disorders recorded in only a few cases like acute renal failure, cystitis or hydrocephalus. CNS symptoms included impaired consciousness to the level of stupor or coma, seizures, cranial nerve deficits, vestibular and cerebellar ataxia. CNS disorders attributed to disease of other organ systems, e.g., otitis or trauma, neoplasia and malformation, were excluded from this group. There was insufficient data to be classified for 112 dogs, and they were excluded from this analysis. The OR for the CNS symptom group was 2.14 (*n* = 957, OR 95% CI 1.06–4.17, *p* = 0.03) than the rest of the analysed population. ELISA positive dogs had 1.97 times greater risk for developing CNS symptoms than ELISA negative (*n* = 957, RR 95% CI 1.19–3.04, *p* = 0.01). There was no significant increase in the number of ELISA positive dogs in other groups (Table 2). 

A multivariable logistic regression model was used to assess interactions between dog age, sex, and CNS symptoms. Interaction between dog age and sex, sex and CNS symptoms and age, and gender and CNS symptoms were not significant and were not implemented in the model. The interaction between age and CNS was significant at 6 and 11 years (Supplementary Table S3).

**Figure 2:**
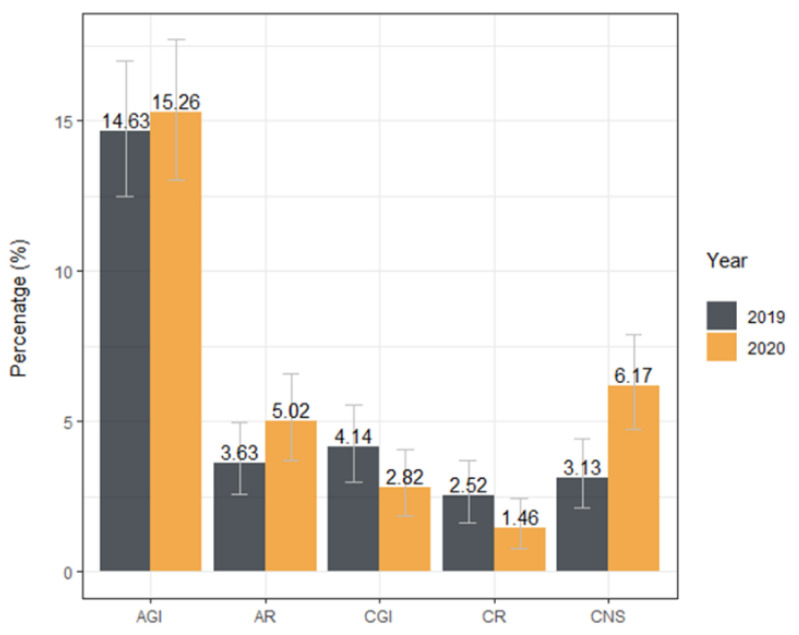
Prevalence of specific diagnosis at dogs admitted to the Veterinary Teaching Hospital in 2019 and 2020. Other diagnoses are not shown in this figure. Error bars represent a 95% confidence interval. AGI—acute gastrointestinal disorders, AR—acute respiratory disorders, CGI—chronic gastrointestinal disorders, CR—chronic respiratory disorders, CNS—central nervous system disorders.

As the final step of the statistical analysis, the prevalence of respiratory, gastrointestinal, and CNS disorders were compared between the general population and control group of 1000 dogs admitted to University Hospital in 2019. In the control group, the medical history of four animals did not contain enough data for their classification, so they were excluded from further analysis. There was no significant difference in age (*n* = 2048, MWU *p* = 0.85) and sex structure (*n* = 2062, χ^2^ = 0.64, *p* = 0.42) between populations. On the other hand, clade composition was different (*n* = 2065, Fisher’s exact test *p* = 0.002). Thus, the influence of clade on CNS clinical signs was tested. Logistic regression showed no influence of the clade on the number of dogs diagnosed with CNS disorder neither in 2019 (*n* = 947, *p* = 0.17) nor 2020 (*n* = 914, *p* = 0.17). In 2020 number of cases that fall into the CNS symptom group more than doubled compared to 2019 (*n* = 1948, OR = 2.03 95% CI 1.3–3.17, *p* = 0.001, RR = 1.97 95% CI 1.29–3.02, *p* = 0.001) (Figure 2). 

## 4. Discussion

The results of this study were heavily dependent on the accuracy of the used serological assays. The previously described specificity of the neutralisation test [6,17,18] was confirmed. To ensure high specificity of ELISA, recombinant purified RBD was used as antigen and corrected OD was calculated. According to available data, this provides higher specificity of the ELISA method in detecting SARS-CoV-2 antibodies [18,19]. The ELISA specificity in the presented study was 100%. Still, false-positive ELISA results could not be completely ruled out. For instance, even though control serum samples were pretested for the alphacoronavirus antibodies, the distinction between CCoV I and CCoV II was not made, and crossreactivity should not be dismissed entirely. It is possible that a larger control group would result in lower specificity of ELISA. There was a discrepancy between ELISA positive samples that tested MNT positive in the general population and COVID-19 infected households. The same was already observed in previous studies [20,21], and there are several possible explanations. Aside from the possible false-positive ELISA results, experimental studies recorded a lower titre of neutralisation antibodies in dogs than in other species [4]. The difference in serological reactivity is further confirmed by comparing serological testing results between dogs and human samples. In December 2020, 458 human serum samples were collected in Zagreb as a part of the general population serosurvey [22]. Serological tests were done by the same research group as in this study, using the same methods in the same laboratory settings. In ELISA reactive samples, neutralising antibodies were detected in significantly fewer dogs than humans (Table 3). The second possibility is that mild infections did not elicit the production of neutralising antibodies, as described in humans [23], or that sampling took place early into an infection or a long time after. The time of virus exposure for dogs in the general population was unknown, and seroprevalence shows a cumulative number of cases. 

On the other hand, almost all dogs from COVID-19 infected households were sampled within four months of the confirmed human case, so it is highly possible that neutralising antibody titre had declined to an undetectable level in dogs infected early into the pandemic. This conclusion is corroborated by significantly more MNT positive dogs at the end of the study period, presumably due to recent exposure. When SARS-CoV-2 seroprevalence in the human and dog population was compared, the difference in sensitivity and specificity of the used serological test is another possible reason behind obtained results. 

Since the beginning of COVID-19 pandemics, SARS-CoV-2 natural infections among dogs were described as isolated cases [24]. Due to higher susceptibility to SARS-CoV-2 infection in experimental conditions and their role in the SARS-CoV epidemic in 2003 [25], cats have been the focus of scientific interest [17,24,26,27]. Only when the first serology results of dogs living in COVID-19 infected households became available, it was clear that human-dog transmissions are far more common than initially thought [7,8,28]. With 43.9% of ELISA and 25.64% of MNT positive dogs in COVID-19 infected households, the results of this study are in line with the studies mentioned above. More importantly, the secondary attack rate for dogs in this study was not different from those recorded in humans [29,30]. Spillover of SARS-CoV-2 to dogs was also visible in high seroprevalence in the general population. In this study, 14.69% of dogs had evidence of SARS-CoV-2 infection, with almost one in five dogs testing ELISA positive at the end of the study period. Those numbers are much higher than those at the end of the first wave in Croatia and other countries [6,7,19]. The most recent serosurvey in France also recorded a high prevalence of ELISA positive dogs (11.1%) in Bouches-du-Rhône, the region with a high number of human cases [31]. Little is known about the dynamic of immune response to SARS-CoV infection in dogs. However, a remarkable increase in the number of MNT positive dogs in the last month of the study suggests recent exposure. The extent of SARS-CoV-2 spread among dogs during the second wave of the COVID-19 pandemic is illustrated when seroprevalence in this study is compared with the earlier mentioned Croatian serosurvey results in the human population. There was no significant difference in the prevalence of ELISA positive samples in the humans and dogs sampled at the same period (Table 3). 

In our study, male dogs in the general population were significantly at increased risk of testing ELISA positive. Increased seropositivity in male dogs was described earlier [7], but was not confirmed in the second study [8]. Both of those studies were conducted on a much smaller sample. Fewer animals and the overrepresentation of female dogs are possible explanations for why male sex was not a significant risk factor in infected households in this research. Many studies show higher COVID-19 susceptibility, severity, and fatality in men [32,33], while others do not [34,35]. In human cases, behavioural and social factors influence the sex distribution of human cases [36]. There is no evidence of SARS-CoV-2 infection independently spreading in the dog population [24]. This study further confirms this observation since dogs in animal shelters were not at increased risk of infection. The difference in social behaviour is a less possible explanation for the observed increased susceptibility in male dogs. It is most probably a result of intrinsic factors described in the human population and animal models [37,38,39,40]. In line with this conclusion is the age distribution of ELISA positive dogs in the general population. Male dogs at reproductive age [37] were significantly more often tested positive than males of any other age or females.

Age was a risk factor in COVID-19 positive households, with a ratio of ELISA positive dogs increasing with age. In the general population, seroprevalence was lowest in dogs less than one year but did not increase with age. Interestingly, a similar age distribution of COVID-19 cases has been observed in the human population [41,42,43]. It has been suggested that a lower number of recorded cases in children could result from lower susceptibility to SARS-CoV-2 infection, milder symptoms of disease or a combination of both [44]. As for sex, in the human population, an age-dependent pattern of SARS-CoV-2 cases may be influenced by extrinsic factors [45]. Well established pathogen, CCoV, has high seroprevalence in the dog population, and there is no difference between age groups [46]. The lower seroprevalence in dogs under the one-year age was described in CRCoV, another betacoronavirus of dogs [11]. The same is true for infections of young horses with equine coronavirus, newly described betacoronavirus [47] but, it is still to be concluded if this age susceptibility is maybe group specific for beta coronaviruses.

A major limitation of any study addressing breed predisposition is their number. In this study, breeds were further grouped based on their phylogenetic relationship [16]. Still, some clades here were represented by few animals or only one breed. Continental Herders were represented by Belgian shepherd dogs but were still only breed clade at increased risk of being infected. Finally, the breed was a significant risk factor, and Belgian shepherd dogs had 3.19 times increased risk of being seropositive. Genetic susceptibility in human cases is drawing much attention [48,49], and the molecular basis of different susceptibility recorded in dogs could give essential answers.

When the COVID-19 pandemic began, symptoms in humans have been suggesting primary respiratory infection, often progressing to pneumonia [1]. With the global spread, it became clear that a substantial number of cases are asymptomatic, or symptoms of other organ system involvement can develop [50]. COVID-19 is now recognised as a multi-system disease with a broad spectrum of clinical manifestations [51]. Dog owners from COVID-19 positive households did not notice significant health issues in their pets. It supports the conclusion that the acute phase of infections in dogs is primarily asymptomatic or mild in their course. There is growing evidence of the long-lasting effects of COVID-19 on human health, even in mild cases. The persistent impact of infection on dog health could not be fully addressed in animals from COVID-19 infected households. Only one animal was sampled more than four months after the assumed infection. Among dogs admitted to the University Hospital, seropositive animals were not at increased risk of respiratory disease or gastroenteritis, as expected based on human or cat infections [52,53,54,55,56].

Contrary to the recent study [57], clinical signs of heart disease were not correlated with serological status. Noticeably, seropositive dogs were almost at the double the risk of developing CNS symptoms. This observation was further supported because, while the prevalence of respiratory and gastrointestinal disorders did not increase in 2020 compared to 2019, CNS disorder prevalence doubled. There is substantial evidence that neural tissue sustains viral replication leading to destruction and altered function [58,59]. Mild-nonspecific neurologic symptoms are observed in human cases [60] that are not clinically assessable in dogs. Severe presentations such as impaired consciousness, acute stroke, seizures, encephalopathy, and meningoencephalitis reported in humans [61,62] are the possible cause of clinical presentation in seropositive dogs presented here since those symptoms can be readily recognised. As opposed to the observed influence of sex on the severity of COVID-19 in humans [63], it was not the case in CNS disorders in dogs.

## 5. Conclusions

There are several limitations of this study. Presented seroprevalence data, as well as risk factor conclusions, are almost solely based on ELISA results. Special effort was made to increase the accuracy of the ELISA used in this research. Corrected OD value was used to diminish the influence of background on ELISA results. Crossreactivity of serum samples was addressed through age stratification and the size of the control group.

Furthermore, control serum samples were pretested for known alpha and beta coronaviruses antibodies. Still, false positive and negative results could not be excluded entirely partially due to limited data on immune response dynamics to SARS-CoV-2 in dogs. The approximate time of infection was known only for dogs in COVID positive households. On the other hand, the sample size in this group was limited, and most of the animals were sampled within four months of the confirmed human case. As a result, conclusions about the sensitivity and specificity of applied serological methods should be taken with caution. Similarly, seroreactivity and the time of infection are indirect or drawn from data available in humans.

Another limitation is the general population sampling. Dogs admitted to Veterinary Teaching Hospital were used to give insights into population dynamics and host factors of SARS-CoV-2 infection in dogs. The study has included a small number of healthy dogs, but some results could be influenced by sampling mostly sick animals. It is reasonable to assume that sick dogs are in closer contact with their owners and veterinary personnel due to the treatment and care they receive. Regarding clinical manifestations, classification was made based on information retrieved from a computer data basis, and additional diagnostic procedures were not plausible. It was not possible to retrieve additional samples, such as cerebrospinal fluid, to confirm virus presence. Highly experienced clinicians made the clinical classification, but it was not easy to make the final decision in some cases. That is why clinical categories are broad, and it is easy to assume that some outcomes of SARS-CoV-2 infection in dos were not captured if they were mild. The study was a blinded, but confirmation bias cannot be excluded. Infectivity and pathogenicity differ between virus variants in humans [64]. Since all dogs were sampled before other variants of concern were confirmed in Croatia [65], additional studies should address potential strain-dependent differences in epidemiology and clinical manifestation of SARS-CoV-2 infections in dogs.

At the moment, there is no indication that dogs have any role as a source of human infections and the virus is spread by human-to-human transmission. This study provides strong evidence that the infection incidence among dogs and humans is not very different. It seems possible that dogs will have a more significant role in the epidemiology of SARS-CoV-2 infections in the future. Recent evidence of infections in pet animals with new SARS-CoV-2 variants [57] raises the possibility that a new virus variant could emerge in dogs as well. Efficient control of SARS-CoV-2 spread in the human population should also include surveillance of dogs and animals.

Finally, there is a strong possibility that dogs are not just being infected, but infection affects their health. It is essential to determine the extent of this effect and risk factors and behaviour to protect dog health without affecting other welfare aspects.

## Figures and Tables

**Table 1:** Monthly seroprevalence rate in dogs admitted to the Veterinary Teaching Hospital from 1 July to 31 December 2020.

	Number of Tested Samples	ELISA ^a^ Positive N (%)	Seroprevalence 95% CI ^b^ (%)	OR ^c^	OR ^c^ 95% CI	*p*
July	126	9 (7.14)	3.32–13.13	0.08	0.04–0.14	-
August	152	23 (15.13)	9.84–21.83	2.32	1.06–5.47	0.042
September	233	46 (19.74)	14.83–25.44	3.2	1.58–7.2	0.002*
October	191	24 (12.57)	8.22–18.12	1.87	0.86–4.38	0.126
November	200	24 (12)	7.84–17.33	1.77	0.82–4.15	0.161
December	167	31 (18.56)	12.97–25.3	2.96	1.41–6.84	0.006 *

Note: Logistic regression was used to calculate the odds ratio with the July rate as the reference category. ^a^ ELISA—enzyme-linked immunoassay, ^b^ CI—confidence interval, ^c^ OR—odds ratio, * statistically significant.

**Table 2:** Association of the serological status on clinical presentation at the Veterinary Teaching Hospital.

Diagnosis	Number of Tested Samples	Number of ELISA ^a^ Positive Samples (%)	Seroprevalence 95% CI ^b^	OR ^c^	OR 95%CI	*p*
Surgical	269	37 (13.75)	9.87–18.46	0.16	0.11–0.22	-
Acute GI ^d^	146	23 (15.75)	10.26–22.69	1.17	0.66–2.05	0.58
Acute respiratory	48	8 (16.67)	7.48–30.22	1.25	0.51–2.77	0.6
Cardio	57	8 (14.04)	6.26–25.79	1.02	0.42–2.23	0.96
Chronic GI ^d^	27	4 (14.81)	4.19–33.73	1.09	0.31–3.03	0.88
Chronic respiratory	14	1 (7.14)	0.18–33.87	0.48	0.03–2.53	0.49
CNS ^e^	59	15 (25.42)	14.98–38.44	2.14	1.06–4.17	0.03*
Dermatologic	77	12 (15.58)	8.32–25.64	1.16	0.55–2.29	0.68
Early infection	31	6 (19.35)	7.45–37.47	1.5	0.53–3.71	0.40
Endocrine	18	1 (5.56)	0.14–27.29	0.37	0.02–1.88	0.34
Healthy	28	4 (14.29)	4.03–32.67	1.05	0.3–2.9	0.94
Malignancy	70	7 (10)	4.12-19-52	0.7	0.27–1.55	0.41
Ophthalmic	18	1 (5.56)	0.14–27.29	0.37	0.02–1.88	0.34
Other	95	8 (8.42)	3.71–15.92	0.58	0.24–1.23	0.18

Note: The most numerous category “Surgical” was used as a reference category in logistic regression analysis. ^a^ ELISA—enzyme-linked immunoassay, ^b^ CI—confidence interval, ^c^ OR—odds ratio, ^d^ GI—gastrointestinal, ^e^ CNS—central nervous system. * statistically significant.

**Table 3:** Seroprevalence among dogs and humans from Zagreb sampled in December 2020.

		ELISA ^a^		Seroprevalence % (95% CI ^b^)	Statistical Significance
		Positive (n)	Negative (n)		
Dog		31	136	18.56 (12.97–25.3)	ELISA positivity rate dog/human *n* = 625 χ2^e^ = 0.184 df = 1 *p* = 0.667
MNT ^c^	Positive	16	NT ^d^	-	
	Negative	15	NT ^d^	-	
Human		94	364	20.52 (16.92–24.52)	MNT positive dog/human samples *n* = 125 χ2^e^ = 4.658 df = 1 *p* = 0.031 *
MNT ^c^	Positive	70	NT ^d^	-	
	Negative	24	NT ^d^	-	

Note: Only ELISA positive samples were tested for neutralisation. Data were analysed using a chi-square test. ^a^ ELISA—enzyme-linked immunoassay, ^b^ CI—confidence interval, ^c^ MNT—microneutralisation test, ^d^ NT—not tested, ^e^ chi-square test value, * statistically significant.

## Data Availability

The authors declare that the data supporting the findings of this study are available within the article and its Supplementary files. Additional information is available from the authors upon reasonable request. Source data are available at doi:https://doi.org/10.6084/m9.figshare.14602902.

## Supplementary Materials

The following are available online at https://www.mdpi.com/article/10.3390/v13081430/s1, Table S1: SARS-CoV-2 ELISA (enzyme-linked immunoassay) and MNT (microneutralisation test) result in different age groups among dogs living in COVID-19 positive households, Figure S1: Age and sex distribution of SARS-CoV-2 ELISA positive dogs, Table S2: Breed predisposition to SARS-CoV-2 infection, Table S3: The interaction between age and CNS symptoms in the logistic regression model.
