# Supplementary material for: The Emergence of SARS-CoV-2 within the Dog Population in Croatia: Host Factors and Clinical Outcome
Source: Viruses. 2021 Jul 22;13(8):1430. doi: 10.3390/v13081430 (PMC8402787; doi:10.3390/v13081430)
Supplement: Supplementary file 1 [file viruses-13-01430-s001.zip › viruses-1272062-supplementary.pdf]

Supplementary data

Table S1. SARS-CoV-2 ELISA (enzyme-linked immunoassay) and MNT (microneutralisation test) result in different age groups among dogs living in COVID-19 positive households.

Age (years)	Number of tested samples	Number of ELISA positive	OR[a] (95% CI[b])	p	Number of MNT[c] positive	OR (95% CI)	p
<1	7	1	0.17 (0.01 – 0.98)	-	1	0.17 (0,01 – 0.98)	-
1 – 4	22	14	10.5 (1.44 – 27.82)	0.04*	7	2.80 (0.37 – 58.21)	0.38
5+	46	18	3.86 (0.59 – 76.05)	0.23	11	1.89 (0.28 – 37.65)	0.58

Note: Logistic regression was used to calculate OR with dogs under one year of age used as the reference category. [a]OR - odds ratio, [b]CI - confidence interval, * - statistically significant

Table S2. Breed predisposition to SARS-CoV-2 infection.

Clades of breeds	Number of tested samples	Number of ELISA[a] positive samples (%)	Seroprevalence 95% CI[b] (%)	OR[c]	OR 95%CI	p
**Mix breed**	312	50 (16.03)	12.13 – 20.58	0.19	0.14 - 0.26	-
**Alpine**	8	1 (12,5)	0,32 – 52,65	1.04	0.11 – 4.91	0.97
**American Toy**	11	0	NA	0.23	0 – 1.78	0.2
**Asian Spitz**	36	5 (13,89)	4,67 – 29,5	0.91	0.31 – 2.2	0.84
**Asian Toy**	60	9 (15)	7,1 – 26,57	0.96	0.43 – 1.96	0.91
**Continental Herder**	23	9 (39,13)	19,71 – 61,46	3.40	1.38 – 8.06	0.01*
**Drover**	12	3 (25)	5,49 – 57,19	1.92	0.47 – 6.28	0.34
**European Mastiff**	114	11 (9,65)	4,92 – 16,61	0.58	0.28 – 1.1	0.1
**New World**	33	5 (15,15)	5,11 – 31,9	1	0.35 – 2.45	0.99
**Pointer Setter**	30	6 (20)	7,71 – 38,57	1.38	0.51 – 3.27	0.5
**Poodle**	100	11 (11)	5,62 – 18,83	0.67	0.32 – 1.28	0.23
**Retriever**	66	8 (12,12)	5,38 – 22,49	0.76	0.33 – 1.57	0.47
**Samoyed**	8	0	NA	0.31	0 – 2.52	0.33
**Scent Hound**	13	1 (7,69)	0,19 – 36,03	0.62	0.07 – 2.67	0.57
**Schnauzer**	19	4 (21,05)	6,05 – 45,57	1.51	0.45 – 4.19	0.48
**Small Spitz**	22	2 (9,09)	1,12 – 29,16	0.63	0.12 – 2.07	0.48
**Spaniel**	37	3 (8,11)	1,7 – 21,91	0.53	0.14 – 1.46	0.24
**Terrier**	78	10 (12, 82)	6,32 – 22,32	0.8	0.37 – 1.57	0.53
**Tibetan terrier**	2	0	NA	1.04	0 – 13.03	0.98
**Toy Spitz**	13	3 (23,08)	5,04 – 53,81	1.73	0.43 – 5.56	0.41
**UK rural**	22	2 (9,09)	1,12 – 19,16	0.63	0.12 – 2.07	0.48

Note: Breeds were grouped in phylogenetic clades [11]. For 72 samples, there was no breed data, and they were excluded from the analysis (n=997). Logistics regression was used to calculate odds ratio values with ″Mix breed″ as reference. [a]ELISA – enzyme-linked immunoassay, [b]CI – confidence interval, [c]OR – odds ratio, * - statistically significant

Table S3. The interaction between age and CNS symptoms in the logistic regression model.

**Age**	**CNS[a] symptoms**				**OR[c]**	**OR 95%CI[d]**	**p (Fisher's exact test)**
	**Present**		**Absent**				
	**No of samples**	**No of ELISA[b] positive**	**No of samples**	**No of ELISA[b] positive**			
**<1**	1	0	46	3	4.14	0.14 – 121.78	1
**1**	7	1	67	12	0.76	0.08 – 6.94	1
**2**	4	0	57	9	0.46	0.02 – 9.11	0.58
**3**	6	1	56	9	1.04	0.11 – 10.03	1
**4**	0	0	51	8	5.12	0.09 – 276.15	1
**5**	4	0	45	11	0.33	0.02 – 6.68	0.56
**6**	7	4	47	8	6.5	1.21 – 34.85	0.04*
**7**	7	1	80	9	1.31	0.14 – 12.2	0.59
**8**	5	0	47	4	0.88	0.04 – 18.62	1
**9**	4	1	64	7	2.71	0.25 – 29.78	0.4
**10**	1	1	72	11	16.04	0.61 – 418.75	0.1
**11**	2	2	81	5	69.55	2.96 – 1634.71	0.01*
**12**	2	1	63	9	6	0.34 – 104,79	0.29
**13**	3	1	37	5	6.4	0.34 – 119.58	0.29
**14**	1	0	39	1	8.56	0.24 – 310.72	1
**15+**	5	2	34	6	3.11	0.42 – 22.87	0.27

Note: [a]CNS – central nervous system, [b]ELISA – enzyme-linked immunoassay, [c]OR- odds ratio, [d]CI - confidence interval.

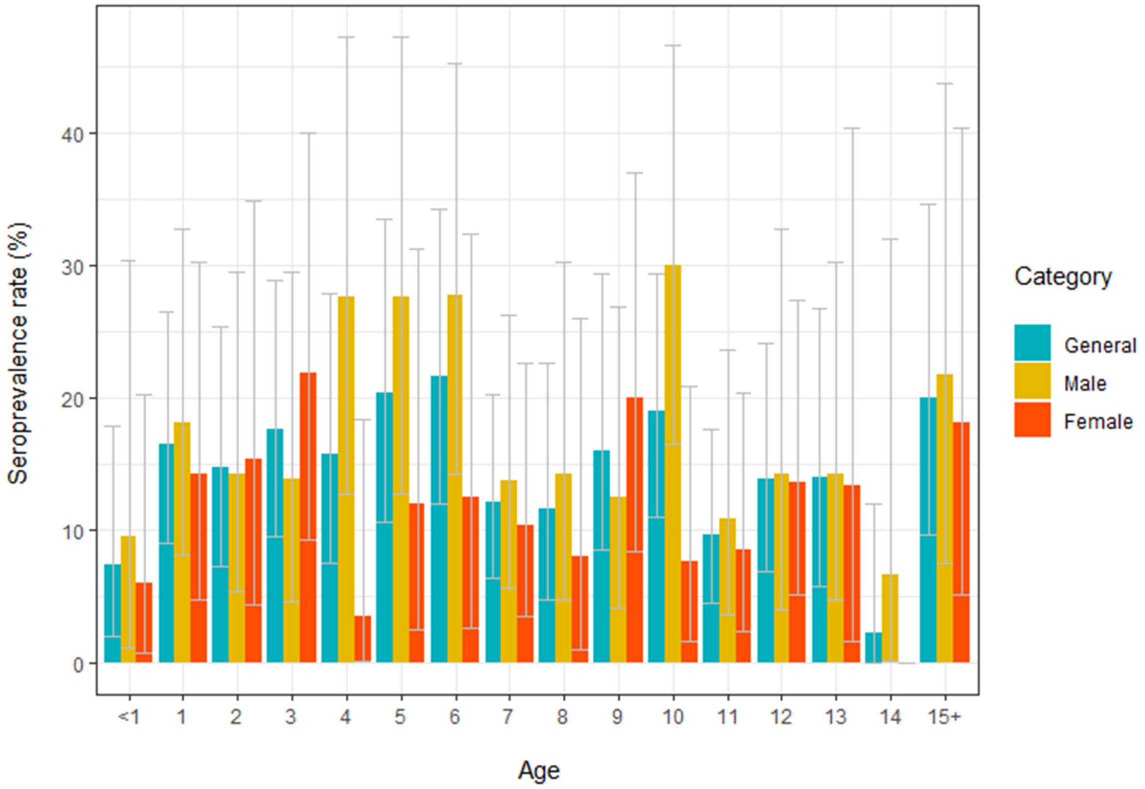

Figure S1. Age and sex distribution of SARS-CoV-2 ELISA positive dogs. Error bars represent a 95% confidence interval.
